# Frankincense extract protects against testicular damage through augmentation of antioxidant defense mechanisms and modulation of apoptotic genes expression

**DOI:** 10.1038/s41598-022-16920-x

**Published:** 2022-07-23

**Authors:** Samir Abdulkarim Alharbi, Mohammed Asad, Kamal Eldin Ahmed Abdelsalam, Sunil Chandy, Monjid Ahmed Ibrahim

**Affiliations:** Department of Clinical Laboratory Science, College of Applied Medical Sciences, Shaqra University, Shaqra, Saudi Arabia

**Keywords:** Infertility, Pharmacodynamics

## Abstract

Frankincense (*Boswellia sacra* Fluck.,) is traditionally used in the treatment of altered male fertile potential in several countries. This study evaluated the cytoprotective action of *B. sacra* oleo gum resin extract against cyclophosphamide (CP) induced testicular toxicity in rats (in-vivo) and lipopolysaccharide (LPS) induced cytotoxicity in human Leydig cells (in-vitro). The methanolic extract of *B. sacra* was standardized for the presence of different boswellic acids using high-performance liquid chromatography (HPLC) and volatile constituents in the extract were detected by gas chromatography–mass spectrometry (GC–MS). Two doses of *B. sacra* extract were used in the in-vivo study. The HPLC analysis showed that extract contains about 36% w/w of total boswellic acids and GC–MS analysis revealed the presence of another 71 different constituents. Administration of *B. sacra* extract to rats increased serum testosterone levels, antioxidant enzyme activities, and sperm count with improved sperm quality in a dose-dependent manner, when compared to CP treated animals. *Boswellia sacra* extract also protected the human Leydig cells against LPS-induced damage and increased the expression of the Bcl-2 gene along with a decrease in caspase-3 gene expression. The results of this study show that *B. sacra* extract has a protective effect on the male reproductive system.

## Introduction

The use of herbs and other supplements for the treatment of altered male fertile potential is increasing throughout the world. There is a growing list of herbal and complementary medicines that are used in the treatment of male sexual dysfunction in different parts of the world while hundreds of herbal and nutritional supplements are available on the market for its treatment^1–3^. The altered male fertile potential is treated by administration of testosterone, nutritional supplements, or antioxidants depending on the cause of the disease^4^.

Frankincense (*Boswellia sacra* Fluck. oleo gum resin) belongs to the family Burseraceae. It is chewed as a mouth freshener in many countries and is also used traditionally for the treatment of digestive, skin, ear, and throat infections, relief of menstrual pain, cardiovascular and neurologic problems, etc., Moreover, products made from *Boswellia* oleo gum resin are marketed throughout the world for various effects^5,6^.

Frankincense is one of the most popular herbs that is used in day-to-day life in many Arab and African countries. One of the reasons for its popularity is that its benefits are stated in the holy texts and several other ancient books. The word ‘frankincense’ is used generally to describe *Boswellia* oleo gum resin and this name is given to several *Boswellia* species that include *B. sacra, B. serrata*, and *B. carteri* depending on the availability of species in a particular country. Most of the chemical constituents in these *Boswellia* species are similar^7^. In Arab countries, the most popular form of *Boswellia* is *B. sacra* locally as ‘*Omani Luban*’ which is used traditionally for the treatment of several diseases and disorders. *Boswellia* species have been investigated for several therapeutic and pharmacological effects. Earlier studies suggest that the most active components in *Boswellia* are the different types of boswellic acids^8^. Some of the therapeutic effects of *Boswellia* species and boswellic acids are antiarthritic, anti-inflammatory, antidiabetic, and anticancer effects apart from their use in the prevention of neurodegenerative diseases. Many of these effects are believed to be due to their potent antioxidant action^9^. The volatile constituents of *Boswellia* are also reported for several effects such as antioxidant, antitumor, anti-inflammatory, and antioxidant actions^10^. We have been investigating *Boswellia sacra* oleo gum resin (*B. sacra*) for different pharmacological and toxicological effects^11–13^. The water extract of *B. sacra* containing volatile constituents aggravated gastric ulcers in rats^11^ while *B. sacra* methanolic extract containing a known amount of boswellic acids was free of hepatotoxic and nephrotoxic effects^12^.

An interesting effect of *Boswellia* species that has not been explored despite traditional claims is the effect on the male reproductive system. It is traditionally used as an aphrodisiac and fertility promoter in Jordan^14^ and frankincense along with other herbs in the form of a mixture is used to treat altered male fertile potential in Iran^15^. Contrary to these effects of frankincense, a study reported that inhalation of ‘*smoke’* by burning *B. papyrifera* and *B. carterii* induces toxic effects on rat testis^16^. However, an earlier study conducted in our laboratory showed that *B. sacra* decreased the expression of *GSTPi, IGFBP3,* and *HSP70* genes with no noticeable changes in the cytoarchitecture of rat testis indicating that it may protect the testis against toxicity^13^.

The present study was a continuation of an earlier study with the standardized methanolic extract of *B. sacra* containing both boswellic acids and volatile constituents. This study evaluated the activity of standardized methanolic extract of *B. sacra* in preventing testicular damage. The effect on different parameters of the male reproductive system in cyclophosphamide (CP) induced testicular toxicity in rats, cell viability, and gene expression in lipopolysaccharide (LPS) induced damage in human Leydig cells were studied.

## Results

### Analysis of the extract

The *B. sacra* extract was found to contain varying amounts of boswellic acids. The amount of boswellic acids ranged from 1.85% w/w of acetyl-11-keto-β-boswellic acid to 23.2% w/w of boswellic acid (α + β). The total boswellic acid is the sum of all the boswellic acid measured in the sample and it was around 36% w/w of the extract (Table 1). About 71 different volatile constituents were detected by GC–MS analysis of the extract (Table 2). The structures of these volatile constituents are given as supplementary material (Table S1).

**Table 1:** Quantity (%) of different boswellic acids.

Type of boswellic acid	Amount (w/w %)
Acetyl-11-keto-β-boswellic acid	1.85
11-keto-β-boswellic acid	6.23
Acetyl boswellic acid (α + β)	7.25
Boswellic acid (α + β)	20.96
Total boswellic acid	36.29

**Table 2:** Name of different chemical constituents detected by GC–MS.

Name of the constituent		
2-Furanmethanol	5-Hydroxymethylfurfural	1,2-ethanediol diacetate
2-Methoxy-4-vinylphenol	Malonic acid, 2-butyl tetradecyl ester	Borane, Diethylmethyl
Formic acid, hex-2-yl ester	Icosanoic acid	4-Cyclopentene-1,3-dione
3-Azetidin-1-yl-propionic acid, methyl ester	Ethyl 1-thio-.alpha.-l-arabinofuranoside	Oxiranemethanol
3-Phenyl-2-thioxopropanoic acid	Hexadecanoic acid, 2-hydroxy-1-(hydroxymethyl)ethyl ester	Acetopropanol
Methyl-6-deoxyhexapyranoside	.beta.-l-Rhamnofuranoside, 5-O-acetyl-thio-octyl	Crotonyl isothiocyanate
2-furanmethanol, 5-ethenyl	Phthalic acid, di(2-propylpentyl) ester	2(3*H*)-Furanone, Dihydro-4-hydroxy -
Benzaldehyde, 2-hydroxy-6-methyl-	Alpha, beta Crotonolacton	2(4*H*)-Benzofuranone, 5,6,7,7a-tetrahydro
2,3-Dihydroxypropyl elaidate	-3(5) D1-1,2,4-triazole-d1	3-Ethoxy-4-hydroxyphenyl acetonitrile
Ethyl (9z,12z)-9,12-octadecadienoate	-1,2-cyclooctanedione	Tetradecanoic acid
Octadecanoic acid, 2,3-dihydroxypropyl ester	Acetic acid, propyl ester	2(4*H*)-Benzofuranone, 5,6,7,7a-tetrahydro-6-hydroxy-4,4,7a-trimethyl-
2,8-Dimethyl-2-(4,8,12-trimethyltridecyl)-6-chromanol	2-Pentadecanone, 6,10,14-trimethyl-	2-Furanmethanol, 5-methyl-
Hexadecanoic acid, methyl ester	1,4-Dioxin, 2,3-dihydro-5,6-dimethyl	2-Furancarboxaldehyde, 5-methyl
Pentadecanoic acid	2,5-Anhydro-1,6-dideoxyhexo-3,4-diulose	2,4-Dihydroxy-2,5-dimethyl-3(2*H*)-furanone
5-Chloro-2,2-dimethylpentanenitrile	2-Pyrrolidinone	Ethanol, 2-[(triethylsilyl)oxy]-
Benzenemethanol, 2,5-dimethoxy-, acetate	2,3-Dihydro-5-hydroxy-6-methyl-4(H)-pyran-4-one	Phenol
beta.-d-mannofuranoside, 1-*O*-(10-undecenyl)-	Pentanal	2-Hydroxy-gamma-butyrolactone
Heptadecanoic acid	2-Butene, 1,4-diethoxy-	2*H*-Pyran-2-one, tetrahydro-3,6-dimethyl
9,12,15-Octadecatrienoic acid,	2-Acetyl-2-hydroxy-.gamma.-butyrolactone	7-Oxa-bicyclo[2.2.1]hept-5-en-2-one
Phytol	2,3-Dihydro-3,5-dihydroxy-6-methyl-4H-pyran-2-one	2-Cyclopenten-1-one, 2-hydroxy
9,12,15-Octadecatrienoic acid, (Z,Z,Z)-	1,1,3,3-Tetramethyl-1,3-bis[3-(2-oxiranylmethoxy)propyl]disiloxane	3-Methylpent-2-ene-1,5-diol
Tricyclo[7.1.0.0[1, 3]]decane-2-carbaldehyde	Silane, [(1,1-dimethyl-2-propenyl)oxy]dimethyl	Proceroside
Octadecanoic acid	1,2-Dioxetane, 3,4,4-trimethyl-3-[[(trimethylsilyl)oxy]methyl]-	Trimethyltetrahydropyran
1,2-Benzenediol	Benzofuran, 2,3-dihydro-	

### Effect on CP induced testicular damage in rats

**Figure 1:**
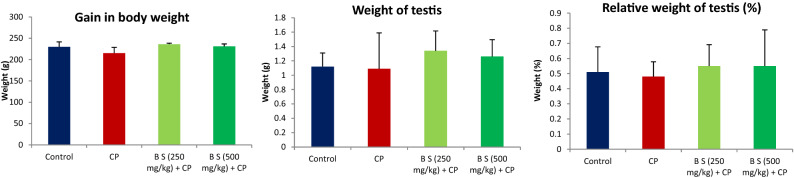
Effect on body weight, testis weight, and relative weight of testis to body weight. All values are mean ± SEM, n = 6. There was no significant change in the weights between the groups. CP = cyclophosphamide (200 mg/kg, i.p), BS *Boswellia sacra extract.*

Administration of *B. sacra* extract for 60 days to rats did not affect their body weight, weight of the testis or relative weight of the testis to body weight (%), when compared to the control or CP treated group (Fig. 1). Similarly, no significant difference in the weight of cauda epididymis was observed (Supplementary data—Fig. S1).

**Figure 2:**
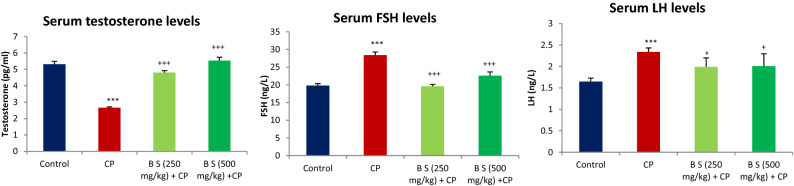
Effect on serum hormone levels. All values are mean ± SEM, n = 6. ***P < 0.001 compared to control. ^+^*P* < 0.05, ^+++^*P* < 0.001 compared to CP treated group CP = cyclophosphamide (200 mg/kg, i.p), B S = *Boswellia sacra* extract.

A significant reduction in the serum total testosterone levels with a feedback increase in the serum levels of FSH and LH was observed in animals that received CP (200 mg/kg, *i.p*) on day 52 of the treatment period when compared to the control animals (*P* < 0.001). The methanolic extract of *B. sacra* at both the tested doses prevented the CP-induced suppression of serum levels of testosterone (*P* < 0.001). The hormone level was similar to that of normal control animals that received only a vehicle indicating that testosterone levels were restored. Attenuation of CP effect on testosterone levels by *B. sacra* extract prevented an associated increase in the serum levels of FSH and LH and these levels were similar to that observed in normal control animals (Fig. 2).

**Figure 3:**
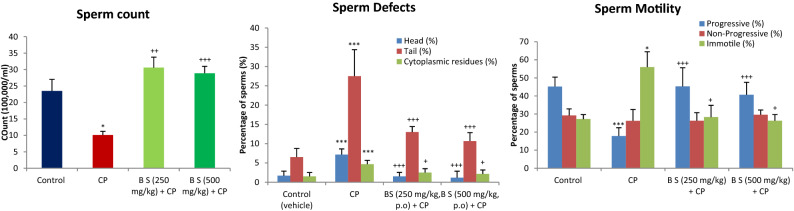
Effect on different sperm parameters. All values are mean ± SEM, n = 6. *P < 0.05, **P < 0.01, ***P < 0.001 compared to control. ^+^*P* < 0.05, ^++^*P* < 0.01, ^+++^*P* < 0.001 compared to CP treated group CP = cyclophosphamide (200 mg/kg, i.p), B S *Boswellia sacra* extract.

**Figure 4:**
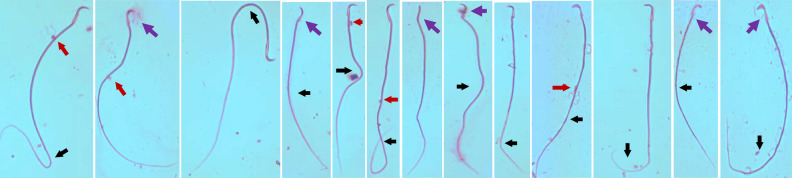
Different sperm defects observed in head and tail. The defects in the head are shown by a purple arrow (), and the tail defects are shown by a black arrow (). Red-colored arrows () show cytoplasmic residues.

A significant decrease in sperm count along with sperm defects in the head, tail, and cytoplasmic residues were observed in CP-treated animals compared to normal control rats (*P* < 0.001). The methanolic extract of *B. sacra* blocked the cytotoxic effect of CP on sperms in a dose-dependent manner as indicated by an increase in sperm count and a decrease in sperm defects. There was no significant difference between sperm defects observed in animals treated with *B. sacra* extract compared to normal control animals (Fig. 3). The sperm head defects observed include straight head, banana head, amorphous heads, and headless sperms while different types of bends were observed in the tail. The cytoplasmic residue mentioned in the figure includes the appearance of residues in both heads and tails of the sperm (Fig. 4).

**Figure 5:**
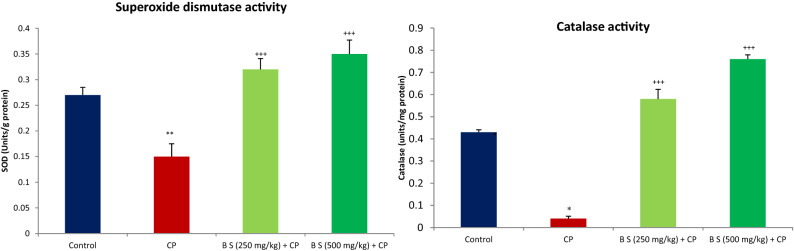
Effect on antioxidant enzymes. All values are mean ± SEM, n = 6. *P < 0.05, **P < 0.01, compared to control. ^+++^*P* < 0.001 compared to CP treated group CP = Cyclophosphamide (200 mg/kg, i.p), B S *Boswellia sacra* extract.

A significant decrease in the activity of antioxidant enzymes was observed due to CP-induced damage when compared to control (Fig. 5). *Boswellia sacra* extract treatment at both doses prevented CP-induced effect on superoxide dismutase (SOD) and catalase activities (*P* < 0.001). There was no significant difference in the activities of these enzymes in *B. sacra* extract-treated animals when compared to control suggesting that enzyme activities were restored to normal.

**Figure 6:**
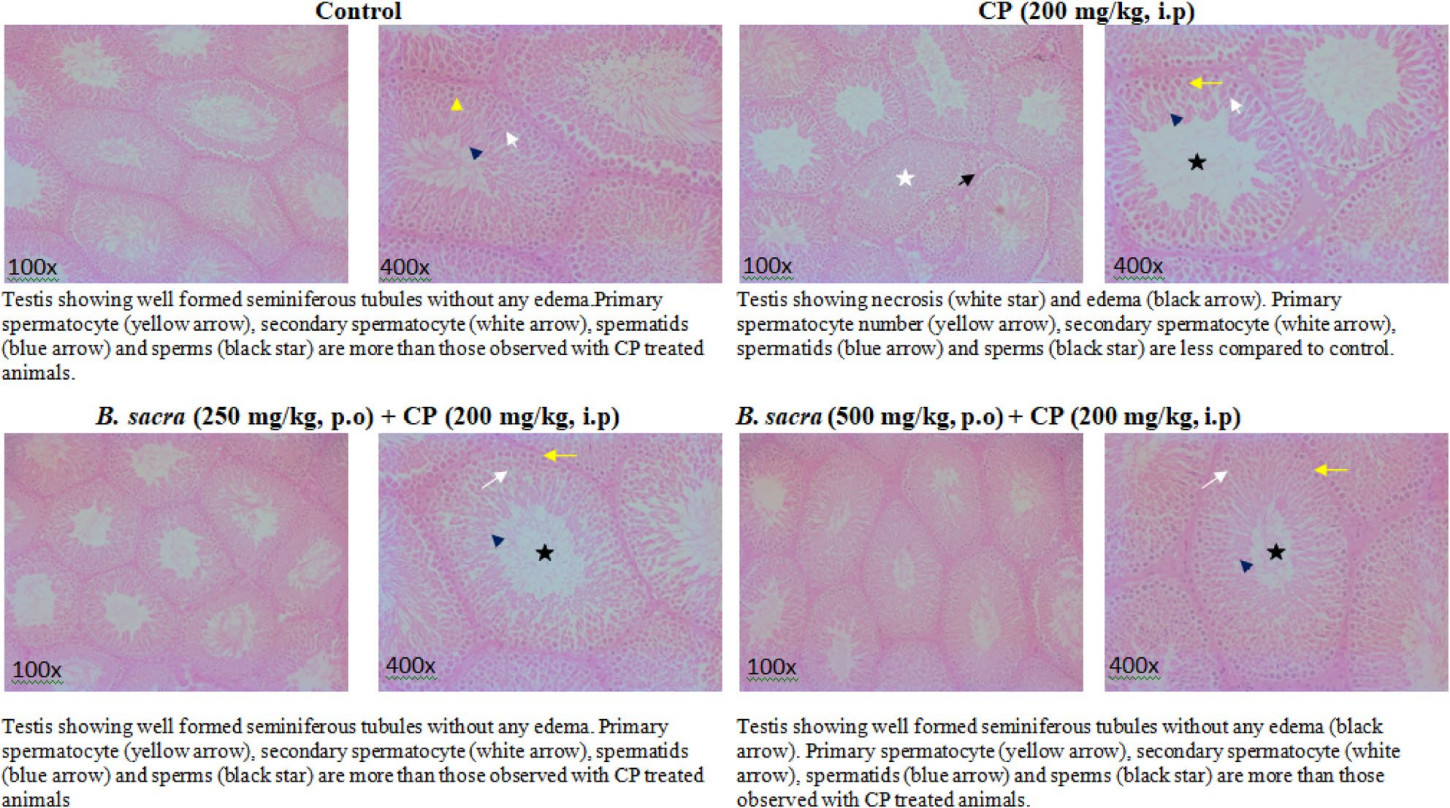
Histological examination of testis.

The effect of different treatments on the seminiferous tubules is shown in Fig. 6. CP administration decreased spermatogenesis by reducing the number of primary spermatocytes along with a reduction in secondary spermatocytes, spermatids, and matured sperms when compared to control. It also produced edema. Pretreatment of animals with *B. sacra* extract for 52 days and further treatment for another 8 days after CP administration was effective in preventing CP-induced testicular toxicity. No abnormal changes in the cytoarchitecture of the seminiferous tubules, number of spermatocytes, and sperms were observed in animals that received either dose of *B. sacra* extract along with CP compared to CP treated animals. The histological characteristics in the *B. sacra* extract-treated animals were similar to control animals.

### Effect on human Leydig cells (in-vitro)

**Figure 7:**
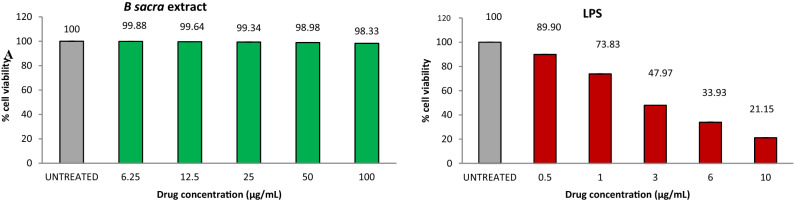
Cell viability assay on human Leydig cells (in-vitro). All values are mean ± SEM, n = 6.

**Figure 8:**
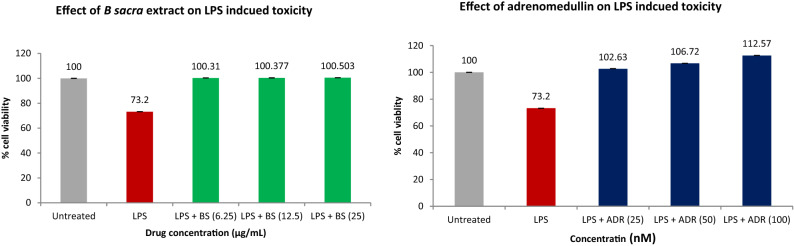
Cytoprotective effect on LPS induced cytotoxicity in human Leydig cells (in-vitro). All values are mean ± SEM, n = 6.

**Figure 9:**
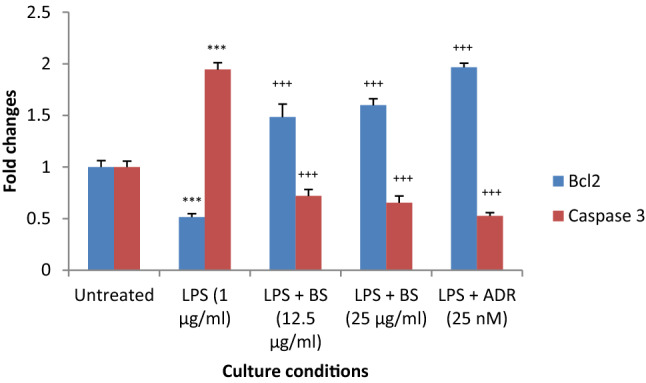
Effect on expression of Bcl-2 and caspase-3 in human Leydig cells (in-vitro*)*. All values are mean ± SEM, n = 6. ***P < 0.001 compared to control, ^+++^*P* < 0.001 compared to LPS treated cells. *B S* *Boswellia sacra* extract, *ADR* Adrenomedullin.

The cell viability of human Leydig cells after incubation of *B. sacra* extract and LPS is shown in Fig. 7. Concentrations of *B. sacra* extract up to 25 µg/mL showed around 99% cell viability while the toxic LPS reduced cell viability depending on the concentration used. The cell viability was reduced to 89.9% at a concentration of 0.5 µg/mL and the percentage of viable cells went down to about 21% at a concentration of 10 µg/mL of LPS. Based on these results, a concentration of 1 µg/mL of LPS and 25 µg/mL of *B. sacra* extract was used to study the cytoprotective effect. At 1 µg/mL of LPS, the cell viability was about 73%, which was above the IC_50_ value and it was ideal to determine the cytoprotective effect of the extract. The cytoprotective assay of *B. sacra* extract in the presence of LPS revealed a dose-dependent effect in preventing LPS-induced cytotoxic damage. However, the effect was less compared to adrenomedullin, which was used as a standard cytoprotective agent (Fig. 8). A study on the expression of the Bcl-2 and caspase-3 genes expression in Leydig cells confirmed the cytoprotective effect of *B. sacra* extract. A significant increase in the expression of the Bcl-2 gene, an antiapoptotic gene along with a significant decrease in the expression apoptotic caspase-3 gene was observed in a concentration-dependent manner after incubation of cells with *B. sacra* extract*.* Similar to the cytoprotective effect, the effect was less compared to adrenomedullin (Fig. 9).

## Discussion

The results of the present study show that *B. sacra* extract possesses a cytoprotective effect on the testis. This action is mediated through enhanced levels of antioxidant enzymes in the testis and an increase in the expression of the anti-apoptotic gene (Bcl-2) and suppression of apoptotic gene (caspase-3) in-vitro. Protection of testis against CP-induced damage by *B. sacra* led to an improvement in sperm count, quality of sperm, and an increase in the secretion of testosterone. In the current study, we administered *B. sacra* extract to 28–30 days old animals and continued the treatment for 60 days. The study protocol was chosen based on earlier reports that damage or protection to the testis is reversible in young animals compared to old animals^17^.

The methanolic extract was used in the current study because methanol is one of the best solvents for the extraction of chemical constituents from herbs^18^. The extract showed the presence of boswellic acids and several volatile constituents. The HPLC analysis was done by dissolving the chemical constituents present in the extract using methanol, as all the constituents easily dissolve in it permitting easy analysis.

Several testicular toxicants are reported in the literature that includes anticancer drugs such as cisplatin, doxorubicin, and CP and metals such as cadmium. We selected CP because it is a very potent testicular toxicant and it is one of the most widely used agents to induce testicular damage to study the protective effect of cytoprotective agents on the testis^19^. CP is known to induce several genetic and biochemical changes in the testes leading to damage. Studies have shown that acrolein, a metabolite of CP induces oxidative stress^20,21^ and the generated reactive oxidative species cannot be mitigated by the limited antioxidant capacity of the testis^20–22^. Only a single dose of CP was given and the changes in the testis were observed after 8 days^22,23^.

The effect of *B. sacra* and CP on the weight of the animals, testis weight, and relative weight of testis to body weight were not significantly different. This observation is similar to earlier studies where a single dose of CP did not significantly affect the body weight or weight of the testes significantly^24^**.**

The testis is vulnerable to damage by reactive oxidative species due to the presence of several factors that includes enzymes such as NADPH oxidase, xanthine oxidase, and mitochondrial electron transport chain^25^. The generation of oxidative radicals is exacerbated by CP and several antioxidants are known to protect testis against CP-induced testicular damage^26,27^. Furthermore, antioxidants are also recommended for the treatment of altered male fertile potential to protect testis from endogenous oxidants and it has been reported that consumption of antioxidant supplements by men may increase the live birth rate in infertile couples^28^. *Boswellia sacra* is a well-known antioxidant and it is known to protect several organs against oxidative damage both in-vivo and in-vitro^29,30^. In the current study, it increased the levels of antioxidant enzymes; the SOD and catalase indicating that it attenuated the CP-induced oxidative damage. As mentioned above, in our earlier study, *B. sacra* extract had decreased the expression of genes involved in the synthesis of antioxidant enzymes in normal animals suggesting that it reduces the level of reactive oxidative species in the testis^13^. Since *Boswellia* species are known to have antioxidant effects in-vitro, the increase in the activities of the antioxidant enzymes in CP-treated animals by *B. sacra* could be due to direct scavenging of reactive oxidative species by the extract than through an increase in expression of the antioxidant gene expression.

CP is known to affect sperm count and sperm motility. This effect is also attributed to the generation of oxygen free radicals that leads to the peroxidation of lipids, oxidation of sulfur-containing proteins, DNA damage, and oxidative stress in mitochondria leading to reduced availability of ATP^31^. The sperm motility is impaired due to the reduced amount of ATP^32^. The toxic effect of CP on sperm count, sperm motility, and sperm defects were prevented by *B. sacra* extract. The effect on sperm quality and the number was supported by histological studies on the testis, where the density of spermatocytes and sperms in *B. sacra* treated rats was more than those treated with CP alone. All the effects on sperms could be due to the antioxidant effect of *B. sacra* extract. This antioxidant action may protect testis against oxidative damage that in turn leads to an increase in sperm production and prevents sperm defects while the effect on the sperm motility could be due to enhanced availability of the ATP due to scavenging of oxidative species in the mitochondria.

The serum testosterone levels were lowered 8 days after CP treatment. This is due to a CP-induced decrease in the activities of enzymes such as 3β-hydroxysteroid dehydrogenase and 17β-hydroxysteroid dehydrogenase, aromatase, and cholesterol side-chain cleavage enzyme-P450cc^33^. This effect is mediated through the abundance of reactive oxidative species generating systems and antioxidants are reported to prevent CP-induced decrease in the serum testosterone levels. However, *B. sacra* extract also produces an increase in the serum total testosterone levels in normal rats^13^. The results of the present study indicate that *B. sacra* extract does not increase the secretion of gonadotrophins from the pituitary gland. Hence, it can be suggested that an increase in the levels of serum total testosterone could be due to both antioxidant effect and stimulation of steroid synthesis in the testis. The restoration of serum testosterone levels to normal was associated with a significant decrease in serum levels of gonadotropins; the FSH, and LH. Testosterone synthesis is stimulated by the LH and an increase in the testosterone secretion stimulates a negative feedback mechanism to prevent the release of the gonadotropin-releasing hormone that in turn decreases the release of FSH and LH from the pituitary gland^34^.

The in-vitro study was carried out to study the effect of *B. sacra* on the human testis. This study also determined if the active constituent(s) involved in protection against the testicular damage acts directly or through metabolites. The results showed that *B. sacra* extract is safe and it maintained the viability of human Leydig cells in concentrations up to 100 µg/mL (98%). LPS is an endotoxin that is used to simulate oxidative testicular damage and to study the effect of cytoprotective agents^35,36^. We used adrenomedullin, a chemical known to prevent LPS-induced damage on human Leydig cells as standard^36,37^. LPS was used at a concentration of 1 µg/mL based on the results of the cell viability assay. The concentration of LPS used in this study is similar to earlier reports on the effect of LPS in human Leydig cells^37^. A cytoprotective effect was produced by *B. sacra* extract in a dose-dependent manner though the effect was less compared to adrenomedullin. The crude extract used in the current study contains several constituents and the active component may be present as a small fraction of the total extract. Hence, a potent effect similar to adrenomedullin could not be observed with the extract. The cytoprotective action was further confirmed by studying the expression of Bcl-2 and caspase-3. The results revealed that apoptosis was prevented by both *B. sacra* extract and adrenomedullin in a dose-dependent manner as indicated by a decrease in the expression of caspase-3 and an increase in the expression of the Bcl-2 gene compare to LPS treated cells.

To conclude, *B. sacra* (frankincense) extract possesses a protective effect in the testis that is mediated at least in part due to its antioxidant action. It prevented CP-induced testicular damage in rats as indicated by an increase in the sperm count, sperm morphology, and sperm motility along with protection of seminiferous tubules. The extract also increased serum testosterone levels. The cytoprotective action was further confirmed by in-vitro studies wherein *B. sacra* extract prevented LPS-induced cell damage in human Leydig cells by modulation of the expression of apoptotic genes.

## Methods

### Chemicals

Analytical grade or HPLC grade chemicals were used. The reagents, chemicals, and diagnostic kits were procured from different suppliers.

### Preparation of methanolic extract of *B. sacra*

Frankincense locally called “*Omani Luban”* was purchased from the local market. The ole gum resin was identified by a botanist through an earlier voucher specimen kept in the institute (SU/CAMS/3/2018). The powdered oleo gum resin was extracted using 90% methanol following the standard procedure of Soxhlation^38^ followed by complete removal of methanol solvent in a rotavapor. The extraction yield was 13.2% w/w of the oleo gum resin. The extract was suspended in water using sodium carboxymethylcellulose (1% w/v).

### Analysis of the extract

The extract was subjected to HPLC for quantification of boswellic acids and GC–MS analysis was used to find the presence of different volatile constituents in the extract.

#### HPLC analysis

The HPLC method has been described in detail earlier^12^. Briefly, the extract and different standard boswellic acids were dissolved in methanol followed by injection into the chromatographic system (Shimadzu). Methanol was used as a solvent because the constituents of the extract dissolved easily in it. The mobile phase was a mixture of 950 mL water and 50 mL acetonitrile with 100% methanol. The detector was set at 210 nm for the detection of boswellic acids (α and β) while the 11-keto-β-boswellic acid and acetyl-11-keto-β-boswellic acid were detected at 247 nm.

#### GC–MS analysis

The given extract was analyzed by GC–MS electron impact ionization (EI) method on GC-17A gas chromatograph (Shimadzu) coupled to a GC–MS QP 5050A Mass Spectrometer (Shimadzu). A fused silica capillary column (30 m × 2.5 mm; 0.25 mm film thickness), coated with DB-5 ms (J&W) was used. The following conditions were used for GC–MS run; Injection temperature: 300 °C, interface temperature: 300 °C, ion source was adjusted to 250 °C, carrier gas: helium (flow rate of 1 ml min^−1^). The analysis was performed following temperature program: 1 min. of isothermal heating at 100 °C followed by heating at 300 °C for 20 min. The mass spectra were recorded at 2 scan sec-1 with a scanning range of 40–850 m/z. Each component was quantified based on peak areas and normalization based on the internal standard.

### Animals

Male rats of Wistar strain aged 28–30 days that were maintained in the institutional animal house were used^39^. Animals were maintained under controlled temperature and humidity with access to rat chow and water. The experimental protocol consisted of standard methods and procedures^39^ with minor modifications that include changes in the duration of treatment and the determination of more parameters. The experimental procedures carried out on the rats followed guidelines given by ARRIVE and the National Committee of BioEthics (Government of Saudi Arabia). The protocol was reviewed and approved by the Ethical Research Committee of Shaqra University (approval number—53/10315).

### Effect on CP induced testicular damage in rats

#### Treatment

Four groups of rats consisting of six animals each were treated as follows; the first group received vehicle (sodium carboxymethylcellulose 1% w/v) for 60 days and served as control. The second group was named CP control and it was administered with the vehicle for 52 days followed by administration of CP (200 mg/kg, i.p)^22^ on day 52, 30 min after vehicle administration. The vehicle treatment was continued for another 8 days after CP administration. The third and fourth groups of animals received a suspension of methanolic extract of *B. sacra* orally at a dose of 250 mg/kg and 500 mg/kg for 52 days respectively^12^. CP (200 mg/kg, i.p) was administered to all rats in these two groups on day 52, 30 min after extract administration. The treatment was given for another 8 days after CP administration. The body weight of the animals was noted every 7 days throughout the treatment period.

On day 60, animals were anesthetized using a cocktail of ketamine (91 mg) and xylazine (9.1 mg) at a dose of 1 mL/kg intraperitoneally^40^. Blood was withdrawn through retro-orbital plexus and serum was used to determine total testosterone^41^, FSH, and LH levels by ELISA^42^. The testes and cauda epididymis were isolated and weight was determined separately. The cauda epididymis was used to determine sperm parameters while the testes were subjected to estimation of antioxidant enzyme activities and histological analysis. The animals were euthanized by giving a further dose of ketamine and xylazine (5 times the anesthetic dose)^40^.

#### Sperm parameters

The isolated cauda epididymis was suspended in 3 mL of Hank’s buffered salt solution (HBSS)^43^. The epididymis was minced in this solution using a scissor to take out the sperms into the HBSS. For determination of sperm motility, a drop of the above suspension on a slide was magnified under 100×. Two hundred sperms were observed. The numbers of progressive, non-progressive, and immotile sperms were counted as per the WHO specifications^44^. The fast progressive and slow progressive were categorized under a single group as progressive sperms^45^. The sperm count was estimated by diluting the sperm suspension with sodium bicarbonate-formalin diluting solution (1:20) to stop the sperm motility^46^. A drop of this was inserted into Neubauer’s chamber and the numbers of sperms in two WBC chambers were counted under 100× magnification to determine the number of sperms/mm^3^ by multiplying the number counted by 100,000. The sperm morphology was studied by preparing a smear of the above suspension. A drop of suspension of cauda epididymis in HBSS was smeared on a clean slide. It was air-dried and then stained with eosin (0.5% w/v) for 5 min followed by air drying^46^. The morphology was studied under 400× to determine the defects in the head and tail. Based on morphology, the spermatozoa were classified into four classes; normal spermatozoa (normal head and normal tail), spermatozoa with abnormal head, spermatozoa with abnormal tail, and spermatozoa with cytoplasmic residues^44^. A total of 200 sperms were observed for morphological defects in each animal. Sperm abnormalities of the mid-region were considered as part of tail abnormality^47^.

#### Testis parameters

Antioxidant enzyme activity and histological studies were carried out. One testis from each animal was homogenized with 0.25% w/v sucrose in phosphate buffer at pH 7.4. The SOD and catalase activities were determined by standard procedures^48,49^.

For histological studies, the left testis was fixed with 10% neutral formalin followed by dehydration and embedding in paraffin wax. The cut sections were stained with hematoxylin and eosin stain. The seminiferous epithelium was examined to identify the effect on spermatogenesis and other morphological changes.

### Effect on human Leydig cells (in-vitro)

#### Cell lines

Human Leydig cells (Cat. # 4510) were acquired from Scien Cell Research Labs (CA, USA) and it was cultivated in cell culture medium-Leydig cell medium (Cat. # 4511, Scien Cell Research Labs, CA, USA) with fetal bovine serum by incubating at 37 °C in 5% CO_2_ atmosphere.

#### MTT viability assay

This was carried out to determine the concentrations of the *B. sacra* extract and LPS on human Leydig cells for use in cytoprotective and gene expression studies. A cell suspension (200 µL) was seeded into a 96-well plate at a cell density of (20,000 cells per well) to grow for 24 h at 37 °C with 5% CO_2_. LPS or *B. sacra* extract at different concentrations were added to these cells and incubated for another 24 h. The spent media was then removed and the MTT (3-(4,5-Dimethylthiazol-2-yl)-2,5-diphenyltetrazolium bromide) reagent was added to a final concentration of 0.5 mg/mL of total volume. The plates were protected from light and incubated for 3 h. The MTT was removed and 100 µL of dimethylsulfoxide (DMSO); a solubilizing agent was added with gentle stirring and pipetting up and down to dissolve the MTT. The absorbance was read at 570 nm wavelength to determine cell viability^50^.

#### Cytoprotective effect of B. sacra extract

A similar procedure as mentioned above was followed. Cell suspension (200 μL) was seeded in a 6-well plate (20,000 cells per well) and growth was allowed for about 12 h followed by treatment with 1 µg/mL of LPS for 24 h. The spent medium was removed and appropriate concentrations of *B. sacra* extract or adrenomedullin (standard)^36^ were added followed by incubation at 37 °C for 24 h in a 5% CO_2_ atmosphere. The spent media was removed and MTT reagent was added to a final concentration (0.5 mg/mL) followed by incubation for another 3 h avoiding exposure to light. The MTT reagent was then removed and DMSO (100 µL) was added with gentle stirring in a gyratory shaker and occasional pipetting up and down to enhance dissolution. The absorbance was read at 570 nm wavelength^50^.

#### Effect on apoptotic markers

Cell suspension (2000 μL) was seeded in a 6-well plate (1 million cells per well). After 12 h growth, cells were treated with LPS (1 ug/mL) for 24 h. The spent medium was removed and *B. sacra* or adrenomedullin were added at appropriate concentrations followed by incubation for 24 h and removal of spent media as mentioned above. The cells were collected by trypsinization.

The RNA was isolated using a Qiagen RNeasy kit, treated with DNAse (to avoid genomic DNA contamination), and purified. The RNA was quantified by UV–Visible spectroscopy (Qiaexpert) and cDNA was synthesized (Biorad iscript cDNA synthesis kit) using random hexamer + oligodT primers as per the following reaction; 5× Mix 5 µL, nuclease-free water—3 µL, RNA—2 µg in 15 µL, RT enzyme—2 µL. The primers used are given in Table 3. The cDNA cycle was as follows; priming—5 min at 25 °C, RT—20 min at 46 °C, RT inactivation—1 min at 95 °C. Reaction volume for real-time PCR was 25 µL (SYBR mix-12.5 µL, water-9.5 µL, Forward primer 1 µL, Reverse primer 1 µL, cDNA 1 µL). Real-time PCR was performed in a Rotor-gene machine (Qiagen). QuantiFast SYBR green master mix (Qiagen) was used and primers were validated with SYBR reactions for amplification and melt curves. The concentration of primers used in real-time PCR was 200 nM. No primer had self-annealing or self-dimerization property and they had Tm near 60 °C. Reactions were done at 60 °C, PCR initial activation step—5 min at 95 °C, denaturation at 95 °C—10 s, annealing at 60 °C—20 s, extension—20 s at 72 °C. The number of cycles performed was 40 and the average of duplicated reactions was taken for analysis. The ΔΔCt method was used for calculating fold changes.

**Table 3:** Primer sequences.

Gene	Primer sequence	
*Bcl-2*	Sense primer/5′ primer (Forward primer)	5′CATGTGTGTGGAGAGCGTCAAC3′
	Antisense primer/3′ primer (reverse primer)	5′CAGATAGGCACCCAGGGTGAT3′
*Caspase-3*	Sense primer/5′ primer (Forward primer)	5′TATGGTTTTGTGATGTTTGTCC3′
	Antisense primer/3′ primer (reverse primer)	5′TAGATCCAGGGGCATTGTAG3′
*GAPDH*	Sense primer/5′ primer (Forward primer)	5′TGACAACTTTGGTATCGTGGAAG3′
	Antisense primer/3′ primer (reverse primer)	5′CAGTAGAGGCAGGGATGATGTT3′

### Statistical analysis

Values are expressed as mean ± SEM as mentioned in the footnotes. Statistical differences between the groups were analyzed using one-way ANOVA with Tukey’s post-test using SPSS (version 20 for Windows).

## Supplementary Information

Supplementary Table S1.

Supplementary Figure S1.

## Data Availability

The data is already shown in the manuscript. Raw data showing values in the individual animals will be made available on request. The request should be sent to the corresponding author (mohammedasad@rediffmail.com).
