# Supplementary material for: Frankincense extract protects against testicular damage through augmentation of antioxidant defense mechanisms and modulation of apoptotic genes expression
Source: Sci Rep. 2022 Jul 23;12:12625. doi: 10.1038/s41598-022-16920-x (PMC9308809; doi:10.1038/s41598-022-16920-x)
Supplement: Supplementary file 1 — Supplementary Table S1. [file 41598_2022_16920_MOESM1_ESM.docx]

**Table S1:** Structure of constituents detected by GC-MS

| **Number** | **Name of the constituent** | **Structure** |
| --- | --- | --- |
|  | 2-Furanmethanol | 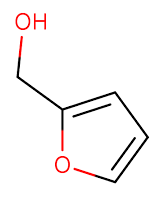 |
|  | 5-Hydroxymethylfurfural | 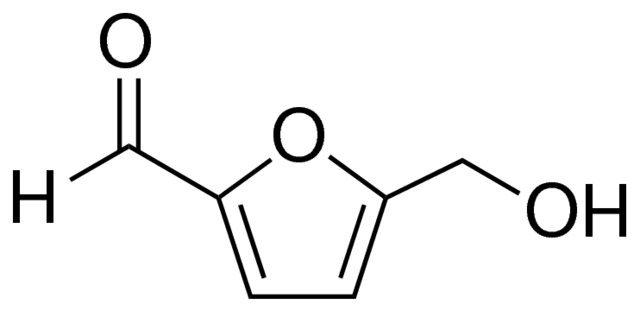 |
|  | 1,2-ethanediol diacetate | 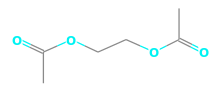 |
|  | 2-methoxy-4-vinylphenol | 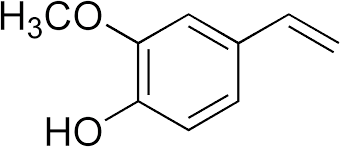 |
|  | Malonic acid, 2-butyl tetradecyl ester | 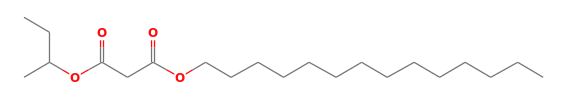 |
|  | Borane, Diethylmethyl | 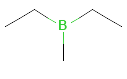 |
|  | Formic acid, hex-2-yl ester | 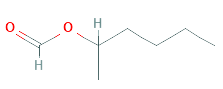 |
|  | Icosanoic acid | 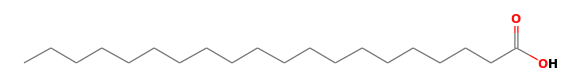 |
|  | 4-Cyclopentene-1,3-dione | 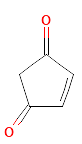 |
|  | 3-Azetidin-1-yl-propionic acid, methyl ester | 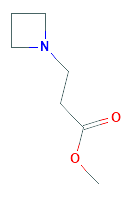 |
|  | Ethyl 1-thio-.alpha.-l-arabinofuranoside | 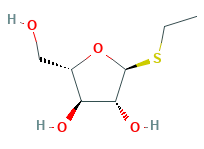 |
|  | Oxiranemethanol | 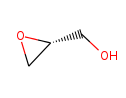 |
|  | 3-phenyl-2-thioxopropanoic acid | 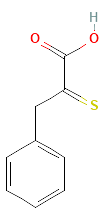 |
|  | Hexadecanoic acid, 2-hydroxy-1-(hydroxymethyl)ethyl ester | 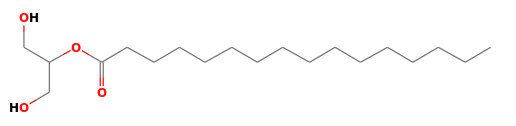 |
|  | Acetopropanol | 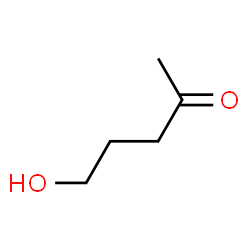 |
|  | Methyl-6-deoxyhexapyranoside | 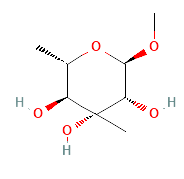 |
|  | .beta.-l-Rhamnofuranosid, 5-O-acetyl-tio-octyl- | 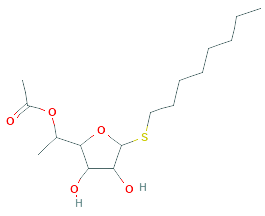 |
|  | Crotonyl isothiocyanate | 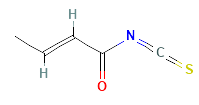 |
|  | 2- furanmethanol, 5-ethenyl | 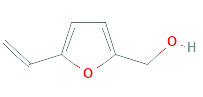 |
|  | Phthalic acid, di(2-propylpentyl) ester | 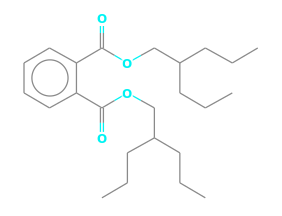 |
|  | 2(3H)-Furanone, Dihydro-4-hydroxy | 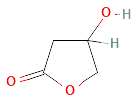 |
|  | Benzaldehyde, 2-hydroxy-6-methyl- | 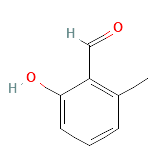 |
|  | Alpha, beta Crotonolacton | 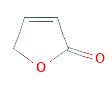 |
|  | 2(4H)-Benzofuranone, 5,6,7,7a-tetrahydro | 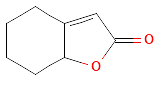 |
|  | 2,3-Dihydroxypropyl elaidate | 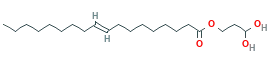 |
|  | 3(5) D1-1,2,4-triazole-d1 | 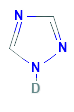 |
|  | 3-Ethoxy-4-hydroxyphenyl acetonitrile | 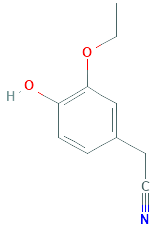 |
|  | Ethyl (9z,12z)-9,12-octadecadienoate | 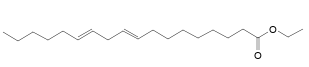 |
|  | 1,2-cyclooctanedione | 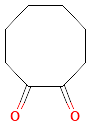 |
|  | Tetradecanoic acid | 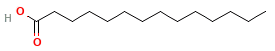 |
|  | Octadecanoic acid, 2,3-dihydroxypropyl ester | 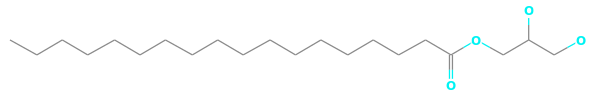 |
|  | Acetic acid, propyl ester | 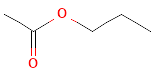 |
|  | 2(4H)-Benzofuranone, 5,6,7,7a-tetrahydro-6-hydroxy-4,4,7a-trimethyl- | 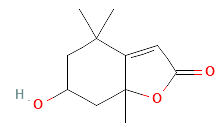 |
|  | 2,8-Dimethyl-2-(4,8,12-trimethyltridecyl)-6-chromanol | 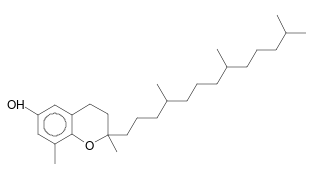 |
|  | 2-Pentadecanone, 6,10,14-trimethyl- | 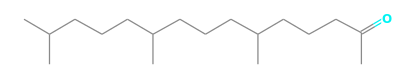 |
|  | 2-Furanmethanol, 5-methyl- | 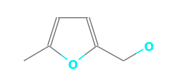 |
|  | Hexadecanoic acid, methyl ester | 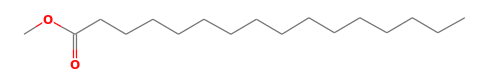 |
|  | 1,4-Dioxin, 2,3-dihydro-5,6-dimethyl-1,4-dioxin | 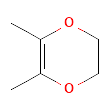 |
|  | 2-Furancarboxaldehyde, 5-methyl | 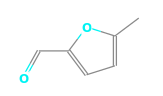 |
|  | Pentadecanoic acid | 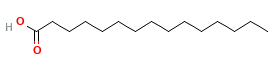 |
|  | 2,5-Anhydro-1,6-dideoxyhexo-3,4-diulose | 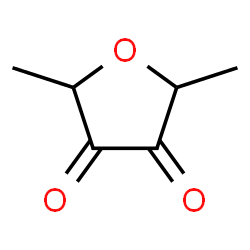 |
|  | 2,4-Dihydroxy-2,5-dimethyl-3(2H)-furanone | 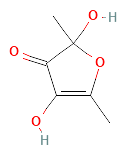 |
|  | 5-Chloro-2,2-dimethylpentanenitrile | 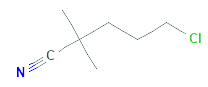 |
|  | 2-Pyrrolidinone | 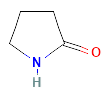 |
|  | Ethanol, 2-[(triethylsilyl)oxy]- | 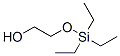 |
|  | Benzenemethanol, 2,5-dimethoxy-, acetate | 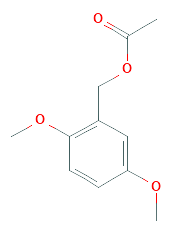 |
|  | 2,3-Dihydro-5-hydroxy-6-methyl-4(H)-pyran-4-one | 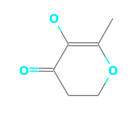 |
|  | Phenol | 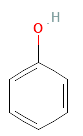 |
|  | beta.-D-mannofuranoside, 1-O-(10-undecenyl)- | 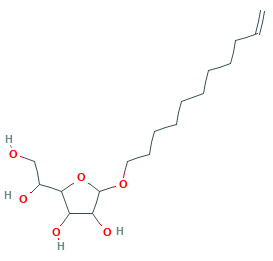 |
|  | Pentanal | 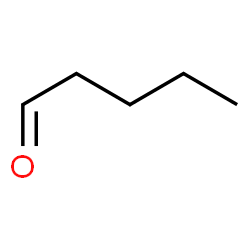 |
|  | 2-Hydroxy-gamma-butyrolactone | 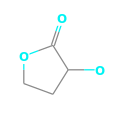 |
|  | Heptadecanoic acid | 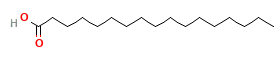 |
|  | 2-Butene, 1,4-diethoxy- | 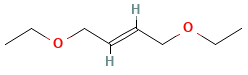 |
|  | 2H-Pyran-2-one, tetrahydro-3,6-dimethyl | 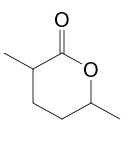 |
|  | 9,12,15-Octadecatrienoic acid | 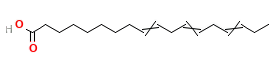 |
|  | 2-Acetyl-2-hydroxy-.gamma.- butyrolactone | 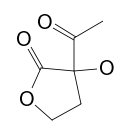 |
|  | 7-Oxa-bicyclo[2.2.1]hept-5-en-2-one | 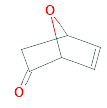 |
|  | Phytol | 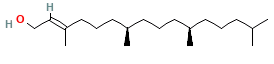 |
|  | 2,3-Dihydro-3,5-dihydroxy-6-methyl-4H-pyran-2-one | 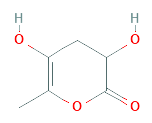 |
|  | 2-Cyclopenten-1-one, 2-hydroxy | 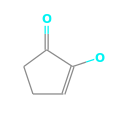 |
|  | 9,12,15-Octadecatrienoic acid, (Z,Z,Z)- | 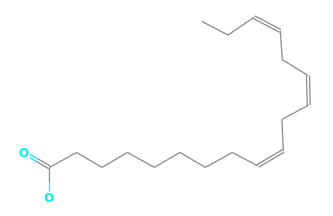 |
|  | 1,1,3,3-Tetramethyl-1,3-bis[3-(2-oxiranylmethoxy)propyl]disiloxane | 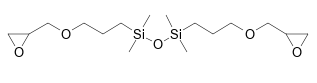 |
|  | 3-Methylpent-2-ene-1,5-diol | 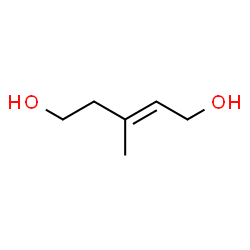 |
|  | Tricyclo[7.1.0.0[1,3]]decane-2-carbaldehyde | 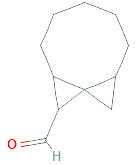 |
|  | Silane, [(1,1-dimethyl-2-propenyl)oxy]dimethyl | 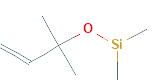 |
|  | Proceroside | 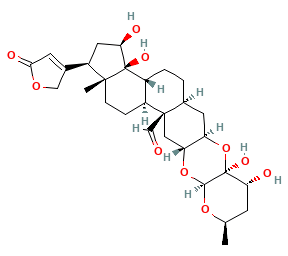 |
|  | Octadecanoic acid | 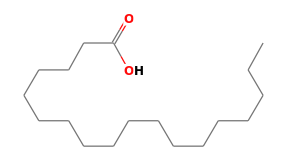 |
|  | 1,2-Dioxetane, 3,4,4-trimethyl-3-[[(trimethylsilyl)oxy]methyl]- | 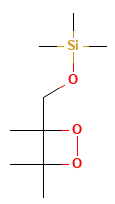 |
|  | Trimethyltetrahydropyran | 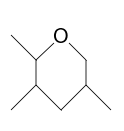 |
|  | 1,2-Benzenediol | 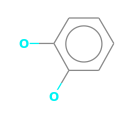 |
|  | Benzofuran, 2,3-dihydro- | 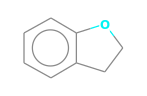 |
