# Supplementary material for: Frankincense extract protects against testicular damage through augmentation of antioxidant defense mechanisms and modulation of apoptotic genes expression
Source: Sci Rep. 2022 Jul 23;12:12625. doi: 10.1038/s41598-022-16920-x (PMC9308809; doi:10.1038/s41598-022-16920-x)
Supplement: Supplementary file 2 — Supplementary Figure S1. [file 41598_2022_16920_MOESM2_ESM.docx]

**Figure S1**: Effect on the weight of epididymis

All values are mean±SEM, n=6. There was no significant change in the weights between the groups. CP = cyclophosphamide (200 mg/kg, i.p), BS = *Boswellia sacra extract.*
